# Quality of sleep in Tunisian patients with Multiple Sclerosis during the COVID-19 pandemic

**DOI:** 10.1192/j.eurpsy.2022.1369

**Published:** 2022-09-01

**Authors:** K. Safa, N. Farhat, S. Salma, B. Nadia, B.A. Mariem, M. Chokri

**Affiliations:** Hbib Bourguiba University, Neurology, sfax, Tunisia

**Keywords:** multiple sclerosis-Quality of sleep-COVID-19 pandemic-patients

## Abstract

**Introduction:**

COVID-19 pandemic has affected human communities around the world, and there is fear that people with chronic diseases such as Multiple sclerosis(MS) are more vulnerable to negative psychological effects.

**Objectives:**

The aim of the present study was to assess the quality of sleep in patients with (MS) in comparison with healthy controls(HCs), during the COVID-19 pandemic and to identify its associated factors.

**Methods:**

This was a cross-sectional survey study conducted with patients followed at the neurology department of Hbib bourguiba university hospital in sfax(Tunisia), during the month of november2020. Sleep quality was evaluated using the Pittsburgh Sleep Quality Index(PSQI). The PSQI is a questionnaire assessing participants’ sleep quality, sleep duration, and sleep disturbances and their severity during the past month.Participants with a PSQI ≤5 are classified as ‘good sleepers’.

**Results:**

Fifty two patients were included in the study. The mean age was 33.69 years(SD=9.21 years)and the sex ratio(F/H)was 4.77. Overall, our patients had higher scores of(PSQI)compared to HC and these différences were statistically significant(p < 0.05). The mean score of(PSQI)was 11.04(SD=3.003)and 11.53%were classified as ‘good sleepers’. Unemployment was related to a poor sleep quality(p=0.0001). Patients with high EDSS(r=0.7;p=0.0001), high number of relapses(r=0.58 ;p=0.0001)were more likely to have sleep disturbance. There was a positive correlation between a poor sleep quality and the duration of disease(r=0.38;p=0.005).

**Conclusions:**

We identified that during the COVID-19pandemic patients with(MS)had a worse sleep quality. The COVID-19pandemic poses a challenge to psychological resilience. More studies are warranted to better understand the long-term consequences of the pandemic on mental health of vulnerable people.

**Disclosure:**

No significant relationships.

